# Effectiveness of a Smartwatch App in Detecting Induced Falls: Observational Study

**DOI:** 10.2196/30121

**Published:** 2022-03-21

**Authors:** Bruce Brew, Steven G Faux, Elizabeth Blanchard

**Affiliations:** 1 Department of Neurology St Vincent's Hospital Sydney Australia; 2 University of New South Wales Sydney Australia; 3 Sacred Heart Rehabilitation Service St Vincent's Hospital Sydney Australia; 4 My Medic Watch Sydney Australia

**Keywords:** falls, smartwatch, app fall detection, accelerometer, inertial sensors, older adult, elderly, old age, smart watch, mobile health, threshold-based algorithm

## Abstract

**Background:**

Older adults are at an increased risk of falls with the consequent impacts on the health of the individual and health expenditure for the population. Smartwatch apps have been developed to detect a fall, but their sensitivity and specificity have not been subjected to blinded assessment nor have the factors that influence the effectiveness of fall detection been fully identified.

**Objective:**

This study aims to assess accuracy metrics for a novel fall detection smartwatch algorithm.

**Methods:**

We performed a cross-sectional study of 22 healthy adults comparing the detection of induced forward, side (left and right), and backward falls and near falls provided by a smartwatch threshold-based algorithm, with a video record of induced falls serving as the gold standard; a blinded assessor compared the two. Three different smartwatches with two different operating systems were used. There were 226 falls: 64 were backward, 51 forward, 55 left sided, and 56 right sided.

**Results:**

The overall smartwatch app sensitivity for falls was 77%, the specificity was 99%, the false-positive rate was 1.7%, and the false-negative rate was 16.4%. The positive and negative predictive values were 98% and 84%, respectively, while the accuracy was 89%. There were 249 near falls: the sensitivity was 89%, the specificity was 100%, there were no false positives, 11% were false negatives, the positive predictive value was 100%, the false-negative predictive value was 83%, and the accuracy was 93%.

**Conclusions:**

Falls were more likely to be detected if the fall was on the same side as the wrist with the smartwatch. There was a trend toward some smartwatches and operating systems having superior sensitivity, but these did not reach statistical significance. The effectiveness data and modifying factors pertaining to this smartwatch app can serve as a reference point for other similar smartwatch apps.

## Introduction

The risk of falling increases with age. Approximately 30% of people older than 65 years and living in the community have a fall at least once a year, with an increase of 5% each year [1]. The incidence is even higher in those living in aged care facilities [2]. This is a major public health problem leading to injuries [1,3], loss of quality of life [1,3], loss of independence [1], placement in assisted-living facilities [4,5] and premature mortality [3]. Fall-related injuries represent 21% of the total health care expenses due to injuries [3] and between 0.85% and 1.5% of the total health care expenditure [6]. Lying on the floor for a long time after a fall has been associated with serious consequences, with a greater likelihood of hospitalization, decline in activities of daily living, placement into long-term care, and mortality [4,5].

Assistive technologies such as call alarm systems and personal emergency response systems are increasingly available. This also holds true for wearables, defined as devices that can be worn or are in contact with human skin to continuously and closely monitor an individual’s activities without interrupting or limiting the users’ motions [7]. These are cost-effective in reducing hospital admissions when used within emergency response systems [8,9]. However, these systems are not always used by consumers, in part, due to difficulties activating them, including cognitive impairment at the time of, or prior to, the fall [5].

There is an increasing interest in using sensor systems embedded in smartwatches for health care purposes [10,11]. This is particularly the case with falls detection. Although there are several fall detection devices and apps, none to our knowledge have been subjected to a blinded study to evaluate effectiveness, particularly with a variety of smartwatches and smartphones using different operating systems. This study aims to address these issues.

## Methods

### Ethics Committee

The procedures followed in this study were conducted according to the principles of the World Medical Association Declaration of Helsinki and were approved by the University of New South Wales and St Vincent’s Hospital Human Research Ethics Committee jointly (16/229). The study was independently audited.

### Study Design

This is a cross-sectional blinded study comparing the fall detection classification provided by a smartwatch algorithm with a reference standard’s classification, in this case, a video record of induced falls.

### Participants

A total of 22 volunteer participants deemed to be medically healthy were recruited after satisfying all the inclusion and exclusion criteria. Participants were recruited by distribution of a leaflet on the university campus and compensated for their time. The inclusion criteria were males/females older than 18 years willing and able to provide written informed consent prior to initiation of any study-related procedures. Participants were excluded if they had any of the following: disability that may prevent them from completing the study (eg, severe illness), being suspected of or having a known allergy to any components of the smartwatch, having any injury or medical condition that would be adversely affected by an induced fall, and being pregnant.

### Smartwatch Threshold Algorithm

**Figure 1:**
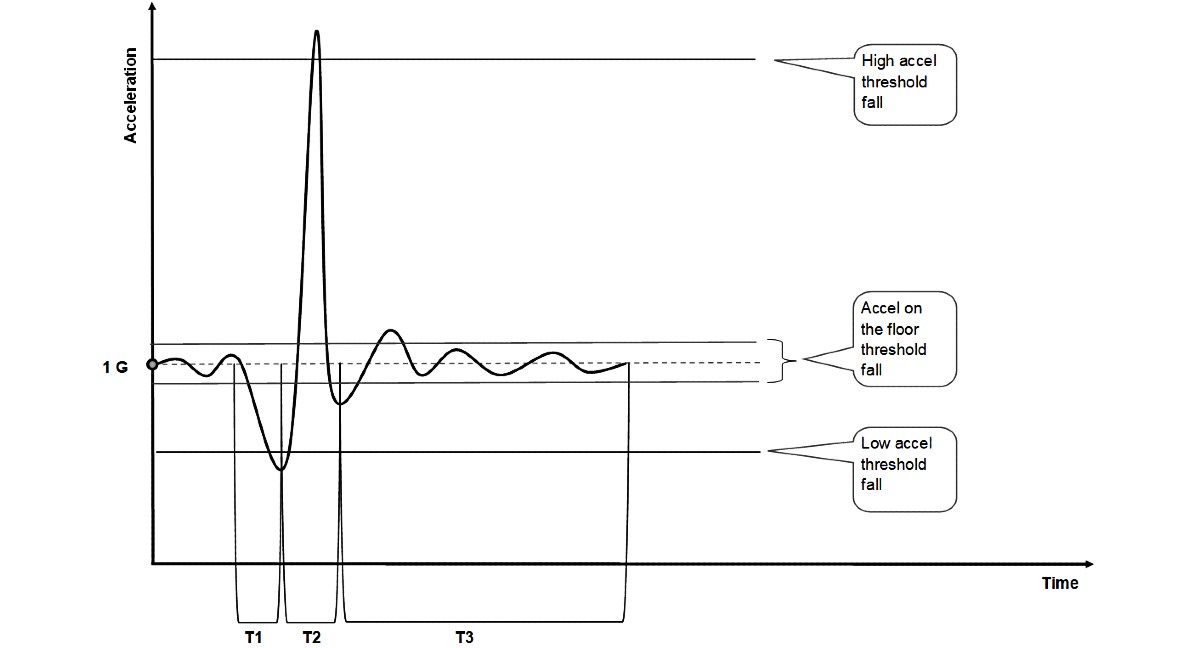
The threshold-based algorithm settings for fall detection. 1G: force of gravity 9.8 m/s^2^; accel: acceleration; T1: time of phase 1 of the fall; T2: time of phase 2 of the fall; T3: time of phase 3 of the fall.

This study used a threshold-based algorithm programmed for different smartwatches. The threshold-based algorithm running on the smartwatch app uses threshold values, or settings, to automatically detect a fall. The frequency of the smartwatch accelerometer is 2 kHz with the algorithm of the app collecting data every 0.01 seconds. The algorithm follows strict rules for the three phases of a fall, as shown in Figure 1. The algorithm was supplied by My Medic Watch.

T1 is defined as the time during which the smartwatch is moving toward the earth (fall time) recording a low acceleration, lower than 1G. T2 is the time during which the smartwatch hits the ground, recording a very high positive acceleration for a short period of time. T3 is the time during which the smartwatch is “almost” immobile on the ground for a long period of time. These threshold values are optimized in the app according to the particular smartwatch and body morphology, including body weight and height. Optimization was performed during the test falls.

**Figure 2:**
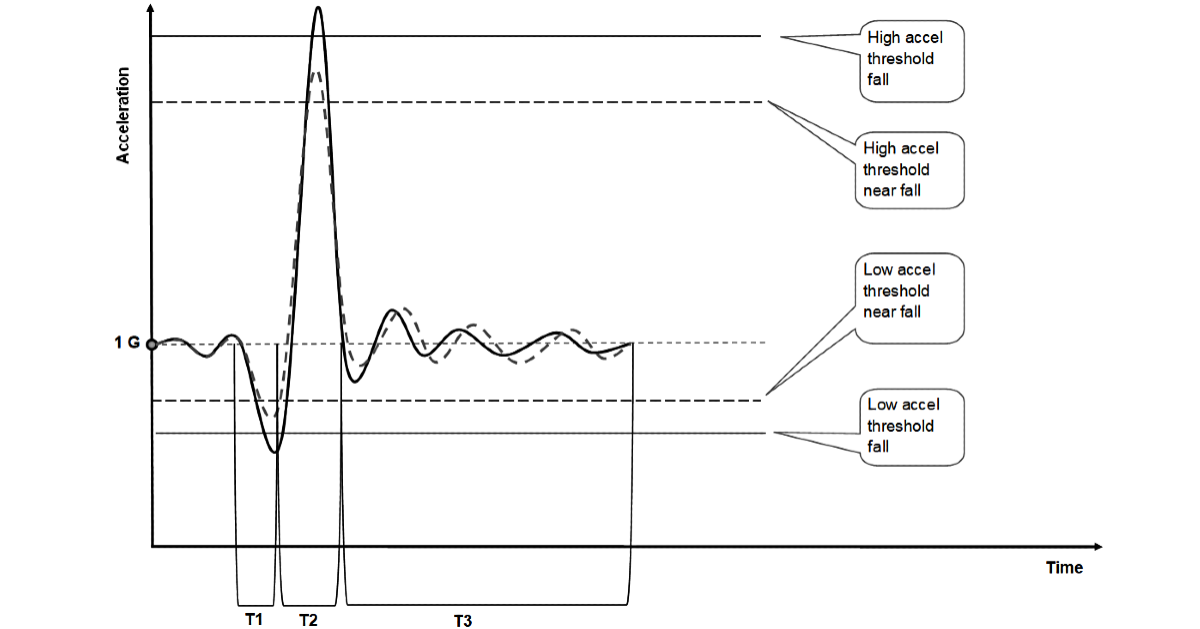
The algorithm threshold settings for the detection of a near fall. 1G: force of gravity 9.8 m/s^2^; accel: acceleration; T1: time of phase 1 of the fall; T2: time of phase 2 of the fall; T3: time of phase 3 of the fall.

A near fall can be recognized when all, or one, of the accelerometer data are close to one of the thresholds, as depicted in Figure 2. We have arbitrarily defined “close” as 20% lower than the fall threshold value.

### Protocol

Participants were randomly assigned to have either smartwatch model A or model C on one wrist and model B or no device on the other wrist. Model A and C were running one operating system, while model B was running on a different operating system. Every smartwatch contained the fall detection app that was programmed to detect and record falls paired with a smartphone located at the study site. The same app was used for each model. The smartwatches and smartphones used one of two operating systems: android or iOS. Two smartwatches were connected to iOS and one to Android. The versions of iOS and Android were the latest available at the time of the test. The version of the operating system on the smartphone and smartwatch were the same for all participants. The smartphones were linked to the smartwatch (according to the operating system) to communicate stored data of the time-stamped recorded episodes to secure cloud servers that were then compared to the video-recorded events.

Before starting the trial, participants were placed in a crash mat protected area, the smartwatches were placed on the participants’ wrists, and a helmet was provided to be used during the tests; no other safety devices were used. Once the trial started, the smartwatch app was set up in monitoring mode and two rounds of four falls were induced in the blindfolded participants. A fall was defined as an event that results in a person coming to rest inadvertently on the ground, floor, or other lower level. A nonfall was defined as any event occurring while both the smartwatch app and the video record were active but excluding a fall or near fall (defined later). In every round, a frontward fall, a right side fall, a left side fall, and a backward fall were induced. These were induced by pushing the participant while standing. The method of fall induction was the same for all participants, executed by the same person. The participants were told of the impending direction of the push. Each assessment took approximately 5 minutes with 8 falls: 2 backward, 2 forward, 2 right, and 2 left. Additionally, up to 3 test falls were performed before the first round to ensure the participants were feeling comfortable with the procedure. Test falls were not included in the analyses. Further, prior to the test falls and between the falls, the participants wore the smartwatches and walked around freely. Near falls where the participant took one or more steps in the direction of the push without falling were also recorded, as there is some evidence that they may presage a fall [12]. This definition is in accord with the traditional definition as applied to this experimental scenario: “a stumble event or loss of balance that would result in a fall if sufficient recovery mechanisms were not activated” [12]. Importantly, the fall-triggering settings were optimized for each participant during the test falls. A non–near fall was defined as any event occurring while both the smartwatch app and the video record were active but excluding a fall or near fall.

During the fall, the algorithm was collecting the acceleration data and the time of the fall. The data collected were in three phases: “prefall” (preparation and walking to the crash mat, several minutes) as soon as the smartwatches were on the participants wrist, “induced fall” (8 falls around 5 minutes), and “post fall,” walking back from the crash matt to the area to remove the smartwatches. In addition to this, the falls were recorded by built-in motion-detecting cameras (recording at 50 frames/second) available at the study site, the National Facility for Human Robot Interaction Research, University of New South Wales. Motion detection data were used to indicate when a fall was observed. The video of the falls also contained a timestamp that was used to compare it with the falls detected by the smartwatch app. In this case, the video recorded event was used as a reference standard, and the falls detected by the smartwatches were compared against it.

After all the falls had been induced, the smartwatches and safety equipment were removed, and participants were observed for approximately 10 minutes: the heart rate, blood pressure, and symptoms (if any) were assessed.

### Data Analysis

To perform the analysis of the falls, data were first retrieved from video records of the built-in motion-detecting cameras and coded as a fall or near fall by the authors and a person independent of the conduct of the study. Where there was disagreement, a majority opinion was taken. These were then compared independently by an external person with data retrieved from a fall detection database built to register the falls detected by the smartwatch algorithm. Each fall was classified as a true positive if the smartwatch app detected a fall at the time when the event was recorded on the video, a false positive if the smartwatch detected a fall event that was not recorded on the video, a false negative if the smartwatch did not detect a fall event recorded on the video, and a true negative if neither the smartwatch nor the video recorded a fall. Near falls were similarly analyzed. Results were computed for sensitivity, specificity, positive likelihood ratio, negative likelihood ratio, positive predictive value, negative predictive value, and accuracy. CIs for sensitivity, specificity, and accuracy are “exact” Clopper-Pearson CIs. CIs for the likelihood ratios are calculated using the “Log method.” To compare fall and near fall detection by smartwatch model and direction of fall only, sensitivity data were used with chi-square tests and a significance value of P<.05. Further data are available on request. Sample size calculations were not formally performed beyond an approximate anticipated number of 20 to 25 participants that could be accommodated for the study given the constraints of the availability of the study site and personnel time.

## Results

### Characteristics of the Participants and the Falls

A total of 22 participants were enrolled in the study: 14 (63%) females and 8 (36%) males; 20 (91%) completed the whole procedure. Two (9%) females abandoned the study during the process: one after a soft tissue injury and the other for unstated reasons. An average of 7.2 falls was performed for each participant; however, one of the participants withdrew from the study after having performed 5 sets of 8 falls, and another after having performed 1 set of 8 falls. Of the induced 226 falls, 64 were backward, 51 were forward, 55 were left sided, and 56 were right sided. Two participants reported postfall self-limiting symptoms associated with soft tissue injuries, 1 required medication and physiotherapy, and their symptoms resolved after 6 weeks.

Demographic characteristics of the participants are shown in Table 1. With regard to BMI, 1 (6%) female was classified as underweight, 1 male and 1 female were classified as overweight (9%), and 1 (6%) male was classified as obese.

**Table 1:** Demographic characteristic of the participants.

Gender	Age (years)	Height (cm)	Weight (kg)
Female	25	160	58
Female	24	167	57
Female	24	170	62
Female	28	153	48
Female	21	164	50
Male	18	177	68
Female	19	164	47
Male	19	175	65
Female	19	164	53
Male	21	174	62
Female	25	170	60
Female	24	163	53
Female	23	168	49
Female	18	174	60
Male	18	180	63
Male	38	171	86
Female	33	163	55
Male	23	184	110
Female	45	160	50
Female	52	163	64
Male	32	160	65
Male	42	178	70

### Overall Performance of the Algorithm

A total of 12 participants were wearing two smartwatches, model A device on one wrist and model B on the other wrist; 10 participants were wearing only one smartwatch, model C, on one wrist. The overall performances of the algorithm, disregarding the model of the smartwatch, are detailed in Tables 2 and 3. There was no difference in the performance of the algorithm according to which wrist if both were used. Tables 4 and 5 represent the results of near fall detection and the associated statistics. The overall test outcomes are summarized in the following section.

In general, the direction of the fall or near fall did not significantly influence sensitivity. Nonetheless, there was a trend for better detection of backward falls: of the 64 backward falls, 11 were false negatives, giving a sensitivity of 82%, versus forward falls, of which there were 51 with 12 false negatives, giving a sensitivity of 76%. Further, there was a significant difference in fall detection if the fall was to the same side versus opposite side of the wrist that had the smartwatch (left sided and right sided sensitivities combined: 92.5% vs 76.3%; P=.009). The same held true for near falls. If the fall was to the same side as the wrist with the smartwatch, there was a 95% sensitivity for left sided falls (55 with 3 false negatives) and 89% sensitivity for right sided falls (56 with 11 false negatives) versus if the fall was on the opposite side as the wrist with the smartwatch, there was 84% sensitivity for left sided falls (55 with 9 false negatives) and 80% sensitivity for right sided falls (56 with 11 false negatives).

**Table 2:** Fall detection results.

True fall status	Test result, n			Total, n
	Negative (nonfall)	Positive (fall)		
Nonfall	265 (true negative)	3 (false positive 1.7%)	268	
Fall	52 (false negative 16.4%)	174 (true positive)	226	
Total	317	177	494	

**Table 3:** Statistics for fall detection.

	Value (95% CI)
Sensitivity (%)	76.99 (70.95-82.31)
Specificity (%)	98.88 (96.76-99.77)
Positive likelihood ratio	68.78 (22.27-212.39)
Negative likelihood ratio	0.23 (0.18-0.30)
Positive predictive value (%)	98.31 (94.95-99.44)
Negative predictive value (%)	83.60 (80.05-86.61)
Accuracy (%)	88.87 (85.76-91.50)

**Table 4:** Near fall detection results.

True near fall status	Test result, n		Total, n
Non–near fall	343 (true negative for all falls, normal falls, and near falls)	0 (false positive)	343
Near fall	43 (false negative when near fall 11.1%)	206 (true positive)	249
Total	386	206	592

**Table 5:** Statistics for near fall detection.

	Value (95% CI)
Sensitivity (%)	88.86 (85.29-91.82)
Specificity (%)	100 (98.23-100)
Positive likelihood ratio	N/A^a^ (no false positives)
Negative likelihood ratio	0.11 (0.08-0.15)
Positive predictive value (%)	100
Negative predictive value (%)	82.73 (78.33-86.39)
Accuracy (%)	92.74 (90.34-94.69)

^a^N/A: not applicable.

### Performance by Smartwatch Model

The number of responses for each smartwatch model were A=186, B=186, and C=122. Model A was used 173 times on the left wrist and 13 times on the right wrist. As per Table 6, there were differences among the models according to sensitivity and specificity, but none were significant. This was also true of the operating system. Similar results were found for near falls.

**Table 6:** Fall detection results by smartwatch models A, B, and C. The direction of the fall did not significantly influence sensitivity in any of the models.

		Value (95% CI)
**Model A**		
	Sensitivity (%)	78.8 (68.6-86.9)
	Specificity (%)	99 (94.6-100)
**Model B**		
	Sensitivity (%)	71.8 (61-81)
	Specificity (%)	98 (93-99.8)
**Model C**		
	Sensitivity (%)	82.1 (96.6-91.1)
	Specificity (%)	100 (94.6-100)

## Discussion

The primary goal of this study was to evaluate the validity of an algorithm programmed in commercially available smartwatches to detect induced falls. Our study found that the algorithm had an overall sensitivity of 77% and specificity of 99%. The false-positive rate was very low at 1.7%, while the false-negative rate was 16.4%. The positive and negative predictive values were 98% and 84%, respectively, while the accuracy was 89%. Falls were more likely to be detected if the fall was on the same side as the wrist with the smartwatch. Similar results were found for near falls. There was a trend toward some smartwatches having superior sensitivity, though neither this nor the operating system reached statistical significance.

Several studies have been conducted to assess the performance of wearable devices for fall detection, mostly by using smartphones or other specialized self-created wearable devices [13-15]. However, only a few of these studies have been performed using commercially available smartwatches [16-19]. In addition, this study is the only one to assess the performance of a fall detection algorithm in different commercially available smartwatches with different operating systems using a video recording system as a gold standard and using blinded data analysis.

The fall detection algorithm was threshold based—programmed to send an alert once a predetermined threshold had been breached. Threshold-based algorithms, as opposed to pattern recognition methods, are preferred on smartphone operating systems due to the restrictions on computing and storage capabilities of the devices [16]. Indeed, pattern recognition methods are costly and need massive analyses of data, access to databases, and long training periods.

Casilari and Oviedo-Jimenez [16] tested different algorithms with an LG W110 smartwatch model R, finding that the fall detection performance depends on the algorithm used. However, there were only 4 participants with a total of 40 falls. Sensitivity ranged from 70% to 100% and specificity from 80% to 100% depending on the type of fall. Mauldin et al [18] have studied three different pattern recognition algorithms based on Naive bayes (NB), support vector machine (SVM), and deep learning models by using a Microsoft band 2 smartwatch. In this context, the algorithm tested in our study performed better than their NB and SVM models in sensitivity and precision, and when compared with their deep learning model, our algorithm performed better in precision but not sensitivity. Mauldin et al [18] also declared in their study that they tested an Android wear-based commercially available fall detection app (Rightminder) released on the Google Play store. The sensitivity was only 50%, and no technical details of this app are publicly available.

Further, these studies have used small groups of participants (3-7) performing several falls each (up to 10 per side). From our experience in laboratory settings, the dynamics of the falls are affected by repetition, as participants tend to fall in the same way. We minimized this effect by having a high number of participants (N=22) repeating each fall only twice per side. Furthermore, the previous studies asked the participants to fall rather than having them fall as a result of being pushed unexpectedly by another person as was done in our study. This approach more accurately reflects a true fall given the spontaneity. The differing protocol designs in these studies make it impossible to accurately compare one against the other.

Our findings suggest that the performance of the algorithm differs among various brand devices. Indeed, the combined performance of brand A and C smartwatches on sensitivity and false-negative rates was higher than the brand B smartwatch. However, the brand B smartwatch precision and thus the false-positive rate is better than brands A and C devices. This is probably related to the differences in the operating systems. Medrano et al [20] explain that in current smartphone operating systems such as Android and iOS, it is difficult to configure specific sampling rates. As the sampling frequencies in both systems are different, the performance of the algorithm will likely be influenced by the operating system used. Moreover, Fudickar et al [21] have investigated the impact of the sampling frequency of the accelerometer on the performance of different threshold-based algorithms in smartphones, concluding that a detection system must deal with the polling frequency of the accelerometer sensors embedded in the device. No studies have been performed regarding this issue on smartwatches; however, it is likely that the situation is the same.

Additionally, our study has found that the performance of the algorithm could be strongly dependent on the smartwatch model. According to Silva et al [22], the performance of a fall detection algorithm could be affected by the quality of the sensors embedded in the device. Additionally, as the manufacturer can change the sensors over time, the performance of the algorithm will also rely on the smartwatch model [16]. This situation could explain the differences we have found between the smartwatch models tested, making it difficult to compare with other studies if they have not used the same smartwatch device and model.

It has been previously reported that the direction of the fall affects the performance of the algorithm used in smartwatches [16,18]. In this context, the performance of the algorithm is largely dependent on which side the fall occurred in relation to the smartwatch. Our algorithm performs better when the fall occurs on the same side of the wrist wearing the smartwatch than when the fall occurs on the opposite side. This is a tendency observed regardless of the smartwatch model. Mauldin et al [18] found a similar performance in the three pattern recognition models they tested. Casilari and Oviedo-Jimenez [16] reported an overall result for side falls; therefore, it is not possible to know if they have found the same tendency.

Regarding the back falls, Mauldin et al [18] found their different algorithm models had poor performance indices in this direction. This was thought to be a consequence of less wrist movement in back falls as compared to other directions of falls. However, our algorithm performed the best on back falls, suggesting that the intensity of the wrist movement or the impact is not affecting the algorithm in this fall direction.

Finally, another factor that could affect the performance of the algorithm in detecting falls in different directions is the participant’s body habitus. It has been proposed that height and weight could affect the performance of the algorithm [23]; thus, implementing personalized settings according to participants’ characteristics is a way to improve the algorithm sensitivity. To address these issues of body habitus and smartwatch model, we deliberately adjusted the algorithm settings during the test falls. This likely contributes to the positive results and should be considered in future studies.

Our study has some limitations. First, there was a relatively small number of participants though not in comparison with other published studies. Second, not all participants wore a smartwatch on each arm, potentially influencing the results. However, only 1 participant was wearing one smartwatch; the results were essentially unchanged with that participant’s data removed. Third, our participants were healthy in contradistinction to the older adult population who would most likely be using the app. Nonetheless, inducing falls in such participants would expose them to considerable risk.

Despite these reservations, the smartwatch app performed well in comparison to studies of other apps and under more rigorous conditions with more stringent analyses, yielding an accuracy of 89%. Indeed, the field of physical activity sensors generally accepts an accuracy of 70% to 80% [24]. Our future research will be focused on investigating the performance of the algorithm in different smartwatch models by using personalized settings. Moreover, head-to-head studies of fall detection devices in smartwatches using real-world participants and settings are likely to improve available evidence concerning the effectiveness of these devices for consumers such as older adults and regulatory or licensing bodies.

## Abbreviations

NB: Naive bayes
SVM: support vector machine
